# The Effect of *Aronia melanocarpa* (Chokeberry) on Body Weight and Fasting Blood Sugar: A Systematic Review and Meta‐Analysis of Randomised Controlled Trials

**DOI:** 10.1002/edm2.70139

**Published:** 2025-11-29

**Authors:** Negin Lohrasbi, Mahsa Elahikhah, Masoomeh Gholizadeh, Farhang Djafari, Nazgol Bahreini, Fatemeh Sheikhhossein, Gholamreza Askari, Mohammad Reza Amini

**Affiliations:** ^1^ Student Research Committee, Department of Clinical Nutrition & Dietetics National Nutrition & Food Technology Research Institute, Shahid Beheshti University of Medical Sciences Tehran Iran; ^2^ Department of Nutrition Sciences, School of Paramedical Sciences Ahvaz Jundishapur University of Medical Sciences Ahvaz Iran; ^3^ Department of Human Nutrition, School of Medicine Urmia University of Medical Sciences Urmia Iran; ^4^ School of Health, Medical and Applied Sciences Central Queensland University Brisbane Australia; ^5^ Department of Nutrition Ahvaz Jundishapur University of Medical Sciences (AJUMS) Ahvaz Iran; ^6^ Department of Clinical Nutrition, School of Nutritional Sciences and Dietetics Tehran University of Medical Sciences (TUMS) Tehran Iran; ^7^ Nutrition and Food Security Research Center Isfahan University of Medical Sciences Isfahan Iran

**Keywords:** body weight, chokeberry, fasting blood sugar, randomised controlled trials

## Abstract

**Background:**

Plant derivatives, particularly anthocyanin‐rich phytochemicals, have been shown to positively influence metabolic parameters. This study aimed to systematically review and meta‐analysis the effects of *Aronia melanocarpa* consumption on body weight and fasting blood sugar (FBS) levels.

**Methods:**

A systematic search was conducted in PubMed/Medline, Scopus, ISI Web of Science, Embase, Cochrane Library, and Google Scholar up to January 2025. Results were reported as weighted mean differences (WMDs) with 95% confidence intervals (CIs) using a random effects model. Heterogeneity among studies was evaluated with the Cochrane Q test and I‐squared (*I* ^2^), and subgroup analysis was performed to identify specific sources of heterogeneity.

**Results:**

Seven studies were identified and included in this meta‐analysis. The findings revealed no significant reductions in FBS (WMDs: 0.05 mmol/L, 95% CI: −0.05 to 0.16, *p* = 0.341), body weight (BW) (WMDs: −0.66 kg, 95% CI: −2.54, 1.22, *p* = 0.494), body mass index (BMI) (WMDs: 0.18 kg/m^2^, 95% CI: −0.17, 0.53, *p* = 0.494), and waist circumference (WC) (WMDs: 0.63 cm, 95% CI: −1.18 to 2.44, *p* = 0.493) in the *Aronia melanocarpa* treatment group. Additionally, subgroup analysis of the included randomised controlled trials showed no significant effects of *Aronia melanocarpa* consumption on BW and BMI.

**Conclusion:**

The present meta‐analysis indicates that Aronia berry consumption does not affect the aforementioned variables. However, further clinical data and research into the mechanisms of *Aronia melanocarpa* are needed to better understand its potential benefits on glycemic markers and anthropometric measures.

## Graphical abstract

Graphical summary illustrating the effects of Aronia melanocarpa (Chokeberry) consumption on metabolic parameters based on a systematic review and meta‐analysis of randomized controlled trials. The analysis showed no significant changes in body weight, BMI, WC, or FBS.

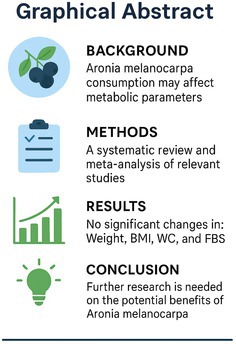

## 1 Introduction

In the early decades of the 20th century, scientific research predominantly focused on exploring foods abundant in phytochemicals and assessing their biological potential. Among the diverse berry family, the *Aronia melanocarpa* , emerged as a particularly intriguing subject of study, primarily due to its exceptional concentration of polyphenolic compounds. Polyphenols, with anthocyanins being a prominent subgroup, demonstrate remarkable antioxidant capabilities [1]. Research suggests that Aronia berries may influence glycemic regulation through unique enzymatic mechanisms. Specifically, studies have demonstrated the berries' ability to inhibit key enzymes α‐glucosidase and dipeptidyl peptidase 4 in both in vitro experiments and in vivo studies using diabetic KKAy mice [2, 3]. Such enzymatic interactions are of interest in developing potential antidiabetic interventions. The inhibition of α‐glucosidase serves to slow the breakdown of disaccharides within the small intestine, consequently delaying glucose absorption into the bloodstream [4]. Meanwhile, dipeptidyl peptidase 4 inhibition prevents the degradation of incretin hormones glucagon‐like peptide‐1 (GLP‐1) and glucose‐dependent insulinotropic polypeptide (GIP) [5]. These hormonal interactions are particularly significant, as GLP‐1 and GIP stimulate insulin secretion, with GLP‐1 additionally reducing glucagon production, ultimately contributing to improved blood glucose management [5]. A study by Milutinović et al. investigating patients with type 2 diabetes mellitus (T2DM) revealed a potential trend toward metabolic improvements following Aronia juice supplementation [6]. However, the results of different studies are contradictory. For example, in the study by Christiane B. et al., even though fermented *Aronia melanocarpa* pulp supplementation led to a higher increase in GIP, no significant effects were noticed in insulin, blood glucose, and GLP‐1 levels [7]. In another study, after a three‐month intervention period, the researchers observed subtle changes in fasting blood glucose and glycated haemoglobin (HbA1c) levels; however, these modifications did not reach statistical significance when comparing baseline and follow‐up measurements [8]. Research has consistently highlighted the potential role of anthocyanins in body weight (BW) management. Investigations suggest that regular dietary intake of these bioactive compounds may contribute to weight control mechanisms, primarily through their demonstrated anti‐obesity characteristics [9]. Moreover, GLP‐1 induces feelings of satiety and has been shown to reduce body weight in animal models of obesity as well as in obese individuals [10]. In an animal model study, oral administration of *Aronia melanocarpa* decreased body weight, fat, and serum lipid profile in obesity‐induced mice without causing histopathological effects [11]. In contrast, in the study by Petrovic et al., after consumption of polyphenol‐rich *Aronia melanocarpa* juice, no differences were observed in anthropometric parameters either before and after the study within the same group or between the *Aronia melanocarpa* and placebo groups [12].

Current understanding suggests an interaction between these bioactive compounds of *Aronia melanocarpa* and metabolic parameters; this systematic review and meta‐analysis aimed to provide an accurate evaluation of the effects of *Aronia melanocarpa* on fasting blood sugar (FBS) and anthropometric indices.

## 2 Methods

The Preferred Reporting Items for Systematic Reviews and Meta‐analysis (PRISMA) guidelines were followed in conducting this systematic review and meta‐analysis [13].

### 2.1 Search Strategy

A systematic search was performed through different medical databases including PubMed/Medline, Scopus, ISI Web of Science, Embase, Cochrane Library, and Google Scholar with no time constraints up to January 2025. Two independent researchers (MRA and FDJ) used the following keywords such as (Chokeberry[tiab] OR aronia[tiab] OR ‘ *aronia melanocarpa* ’[tiab] OR Photinia[Mesh]) AND (‘Body Weight’[tiab] OR ‘Quetelet Index’[tiab] OR ‘Body Mass Index’[tiab] OR ‘Body Weight’[Mesh] OR ‘Body Weight Changes’[Mesh] OR ‘Body Mass Index’[Mesh] OR ‘Weight Loss’[Mesh] OR Obesity[Mesh] OR ‘Waist Circumference’[Mesh] OR ‘Obesity, Abdominal’[Mesh] OR ‘Fasting Blood Sugar’[tiab] OR ‘Blood Glucose’[Mesh]) for search (Table S1). Forward and backward reference searches of included articles were conducted to identify additional studies. This study did not include unpublished manuscripts, conference papers, and thesis.

### 2.2 Eligibility Criteria

To establish the eligibility criteria, the PICOS model [14] was used as follows: population (age > 18 years), intervention ( *Aronia melanocarpa* ), comparator (placebo), outcome (FBS, body mass index (BMI), BW, and waist circumference (WC)) and study design (parallel and cross‐over clinical trials). Studies must meet the following criteria to be included in this meta‐analysis: (a) randomised clinical trials (RCTs) comparing the effect of *Aronia melanocarpa* (chokeberry) to placebo; (b) included adult participants 18 years of age and older; (c) reported results as mean and standard deviation (SD) for FBS, BMI, BW, and WC; and (d) only published in the English language.

Studies were excluded if they had the following exclusion criteria: (a) non‐RCT studies; (b) performed on animals; (c) involved individuals < 18 years old; (d) studies with no placebo group; (e) examined the effect of other interventions along with *Aronia melanocarpa* (chokeberry); and (f) did not report outcomes (FBS, BMI, BW, and WC) at baseline and at the end of the intervention. Books, letters, comments, conference papers, and reviews were also excluded.

### 2.3 Data Extraction

Two investigators (NL and FDJ) independently conducted data extraction for this meta‐analysis, assessed the quality of the included studies, and cross‐verified the results. In cases of disagreement, a third independent investigator (MRA) resolved discrepancies. The data extracted for both the intervention and control groups followed a standardised format and included study characteristics (authors, publication year, study design, country, trial duration, and intervention arms), participant details (inclusion criteria, age, sex, and health status), and assessed outcomes (baseline and final values for BMI, BW, WC, and FBS). For the outcome values, the mean and SD changes were extracted. If a study did not report these changes, the baseline and final values were extracted for both the intervention and control groups. If further information was required, the study authors were contacted.

### 2.4 Quality Assessment

The quality of included studies was independently assessed by two researchers (NL and FDJ) using the Revised Cochrane risk‐of‐bias tool (RoB 1) [15] for randomised trials. The RoB 1 evaluates randomised trials through the following domains: random sequence generation, allocation concealment, blinding of participants and personnel, blinding of outcome assessment, incomplete outcome data, selective reporting, and other potential sources of bias. Following the recommendations outlined in the Cochrane Handbook, studies were categorised as having a low, high, or unknown risk of bias for each domain (Table 1).

**TABLE 1:** Risk of bias for randomised controlled trials, assessed according to the Revised Cochrane risk‐of‐bias tool for randomised trials.

Publications	Random sequence generation	Allocation concealment	Selective reporting	Blinding (participants and personnel)	Blinding (outcome assessment)	Incomplete outcome data	Other source of bias
1. Christiansen (2023)	L	L	L	L	L	L	L
2. Le Sayec (2022)	L	L	L	L	H	L	L
3. Loo (2016)	L	L	L	H	H	L	L
4. Naruszewicz (2007)	L	U	L	L	U	L	L
5. Pokimica (2019)	L	U	L	L	U	L	L
6. Stankiewicz (2023)	L	U	L	L	U	L	H
7. Xie (2017)	L	U	L	L	U	L	L

Abbreviations: H, high risk of bias; L, low risk of bias; U, unknown.

### 2.5 Data Synthesis and Statistical Analysis

The effect of *Aronia melanocarpa* on FBS, BMI, BW, and WC was estimated by pooling baseline and final mean and SD values of the studies in both intervention and control groups. The formula SD^2^ baseline + SD^2^ final −(2 R × SD baseline + SD final) [16] was used to calculate the SD of the mean difference for studies that not reported, where R was considered to be 0.8. Weighted mean differences (WMDs) with 95% confidence intervals (CIs) were calculated to determine the magnitude and direction of the effect, with negative values indicating a reduction in these measures. The 95% CI provides an estimate of precision, where narrower intervals suggest more reliable results. The extent of heterogeneity was assessed using the *I*‐squared (*I* ^2^) statistic, with substantial heterogeneity defined as *I* ^2^ > 50% and a *p*‐value < 0.1 based on Cochrane's test. The Der Simonian and Laird random‐effects model was applied. A subgroup analysis was performed to investigate potential differences in the effects of *Aronia melanocarpa* on BW and BMI based on age, BMI categories, and intervention duration. The fixed‐effects model was used to estimate the pooled effect sizes within each subgroup. Additionally, heterogeneity was assessed using the *I* ^2^ statistic and *p*‐values for subgroup differences to evaluate variability within and between groups. Sensitivity analysis was performed to explore the effect of each study on overall analysis. Additionally, publication bias was assessed using Egger's regression test [17]. Meta‐analysis was conducted using Stata software, version 14 (Stata Corp LP, College Station, TX, USA).

## 3 Results

### 3.1 Study Selection

**FIGURE 1:**
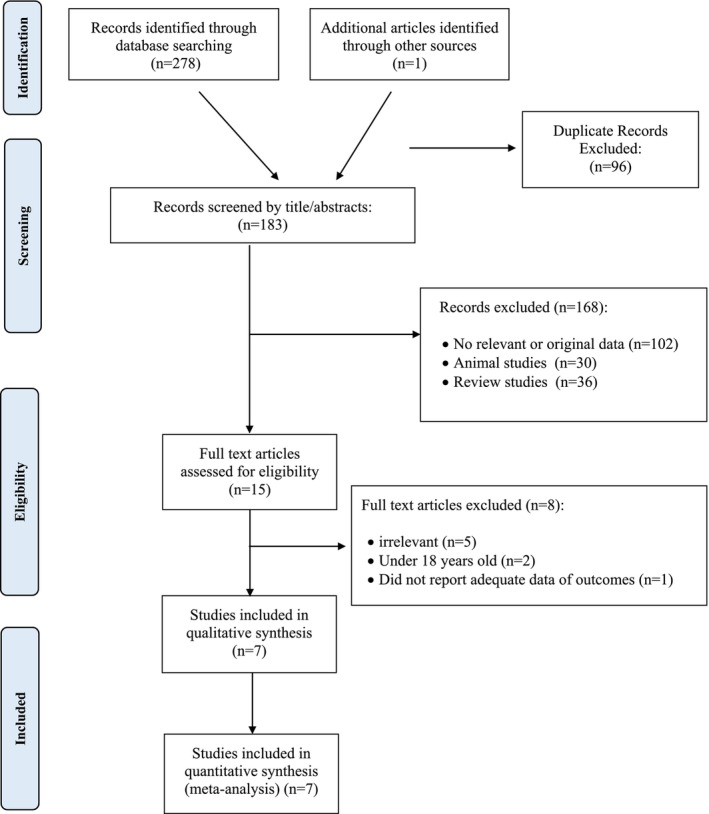
Flow chart of the number of studies identified and selected into the meta‐analysis.

The initial search ended up with 278 articles (Figure 1). When duplicates were removed, 183 articles remained for screening based on title and abstract. 168 articles were excluded, so 15 papers were retrieved for full‐text review. After, careful evaluation, 8 articles were excluded because of the following reasons: (a) irrelevant data (*n* = 5), (b) under 18 years old participants (*n* = 2) [12, 18], and (c) did not report adequate data for the outcomes (*n* = 1) [19]. At the end, seven articles were included in this meta‐analysis.

### 3.2 Study Characteristics

The characteristics of the final studies are shown in Table 2. The sample size varied between 22 [20] and 102 [21]. The participants' mean age ranged between 19.9 [20] and 66.9 [7] years old. The studies were conducted in Poland [20, 22], Denmark [7], The United Kingdom [21], The United States [23], Finland [24], and Serbia [25]. The mean of the baseline BMI for participants was 25.7 kg/m^2^. Studies involved healthy participants [21], former smokers [23], young football players [20], patients after myocardial infarction [22], participants with type 2 diabetes [7], untreated mild hypertension [24], and cardiovascular risk [25]. Studies were published from 2007 to 2023.

**TABLE 2:** Demographic characteristics of the included studies.

First author (year)	Location	Study design	Health status	Sex	Sample size	Duration (week)	Mean age (year)	Baseline BMI (kg/m^2^)	Intervention group	Comparator group	Outcome
1. Christiansen (2023a)	Denmark	RCT, crossover design	Type 2 diabetes	Both	36	8	66.9	28.6	34 g (37%) fermented aronia extract	Placebo	Weight/BMI/WC/FBS
2. Christiansen (2023b)	Denmark	RCT, parallel	Type 2 diabetes	Both	36	8	66.9	28.6	34 g (37%) non‐fermented aronia extract	Placebo	Weight/BMI/WC/FBS
3. Le Sayec (2022)	United Kingdom	RCT, parallel	Healthy	Both	102	12	56.2	24.7	One aronia berry‐extract capsule contained 106 mg total (poly) phenols	Placebo	Weight/BMI/FBS
4. Loo (2016)	Finland	RCT, crossover design	Untreated mild hypertension	Both	37	8	55.8	25.9	300 mL/day chokeberry juice	Placebo	Weight/BMI/FBS/SBP
5. Naruszewicz (2007)	Poland	RCT, crossover	Patients after myocardial infraction	Both	44	6	66	26.7	255 mg Aronia extract	Placebo	BMI/FBS
6. Pokimica (2019)	Serbia	RCT, parallel	Cardiovascular risk	Both	56	4	40.6	27.3	300 ml/day chokeberry juice	Placebo	BMI/FBS
7. Pokimica (a) (2019)	Serbia	RCT, parallel	Cardiovascular risk	Female	31	4	40.6	27.3	300 ml/day chokeberry juice	Placebo	WC
8. Pokimica (b) (2019)	Serbia	RCT, parallel	Cardiovascular risk	Male	25	4	40.6	27.3	300 ml/day chokeberry juice	Placebo	WC
9. Stankiewicz (2023)	Poland	RCT, crossover design	Healthy young footballers	Male	22	13	19.9	20.9	6 g *Aronia melanocarpa*	Placebo	Weight/BMI
10. Xie (2017)	USA	RCT, parallel	Healthy adult former smokers	Both	49	12	35	26.3	500 mg Aronia extract	Placebo	Weight/BMI/WC

Abbreviations: BMI, body mass index; FBS, fasting blood sugar; RCT, randomised controlled trial; WC, waist circumference.

The intervention duration was from 4 weeks [25] to 13 weeks [20]. Four studies [7, 20, 22, 24] had cross over design while the others had parallel design. The different types and dosages of Aronia were applied to the intervention group, including Aronia extract (37 g, 500 mg, and 255 mg), Aronia juice (300 mL of chokeberry), and Aronia berry‐extract capsule (106 mg total polyphenols), and powder (6 g) while the control group received placebo. All the included studies have reported BMI as an outcome. Of them, five articles [7, 20, 21, 23, 24] investigated body weight, five [7, 21, 22, 24, 25] on FBS, and three [7, 23, 25] on WC.

About the quality of the included studies, four studies were not fully clear about their methodology for allocation concealment [20, 22, 23, 25]. One study had a high risk of bias for blinding the participants and personnel [24]. Additionally, two studies [21, 24] showed a high risk of bias and four studies [20, 22, 23, 25] had an unknown risk of bias for blinding the outcome assessment. Also, one study had a high risk of bias for other sources [20] (Table 1).

### 3.3 Effect of *Aronia melanocarpa* (Chokeberry) on BW

**FIGURE 2:**
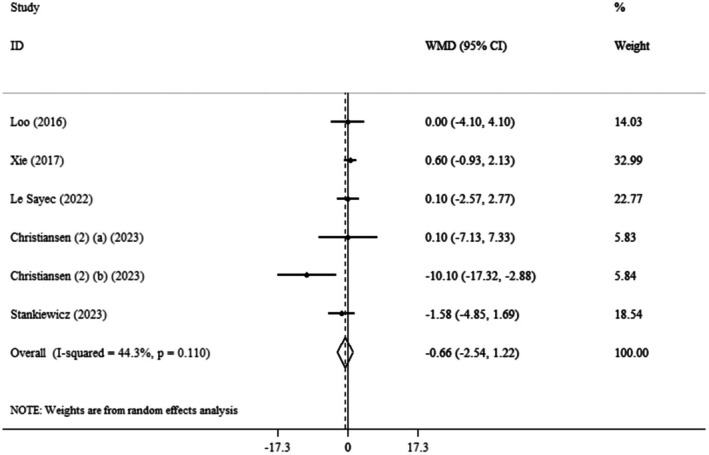
Forest plot detailing weighted mean difference and 95% confidence intervals (CIs) for the effect of *Aronia melanocarpa* (chokeberry) consumption on weight.

The pooled mean effect size was not significant (WMD: −0.66 kg, 95% CI: −2.54, 1.22, *p* = 0.494) (*I* ^2^ = 44.3%, *p* = 0.110) compared to the control group (Figure 2). Also, subgroup analysis showed no significant differences in body weight changes with *Aronia melanocarpa* (chokeberry) across age groups, BMI categories, or intervention durations (Table 3).

**TABLE 3:** Subgroup analysis of included randomised controlled trials in meta‐analysis of the effect of *Aronia melanocarpa* (chokeberry) consumption on weight and BMI.

Group	No. of effect size	WMD (95% CI)	*p*	*I* ^2^ (%)	P‐heterogeneity	*p* for between subgroup heterogeneity
*Weight*						
**Duration (week)**						
≤ 8	3	−1.96 (−5.16, 1.24)	0.23	67.0	0.05	0.22
> 8	3	0.19 (−1.04, 1.42)	0.76	0.0	0.49	
**Age**						
≤ 50	2	0.21 (−1.17, 1.59)	0.77	28.7	0.23	0.45
> 50	4	−0.75 (−2.80, 1.30)	0.47	57.2	0.07	
**Mean BMI**						
< 25	2	−0.57 (−2.64, 1.50)	0.59	0.0	0.44	0.58
25–29.9	4	0.12 (−1.25, 1.50)	0.86	62.8	0.04	
*BMI*						
**Duration (week)**						
≤ 8	5	0.30 (−0.19, 0.79)	0.23	0.0	0.63	0.50
> 8	3	0.06 (−0.43, 0.56)	0.80	34.9	0.21	
**Age**						
≤ 50	3	−0.04 (−0.62, 0.54)	0.89	41.3	0.18	0.34
> 50	5	0.31 (−0.13, 0.74)	0.16	0.0	0.78	
**Mean BMI**						
< 25	2	−0.15 (−0.72, 0.42)	0.60	0.0	0.34	0.14
25–29.9	6	0.38 (−0.06, 0.82)	0.09	0.0	0.69	

Abbreviations: BMI, body mass index; WMD, weight mean difference.

### 3.4 Effect of *Aronia melanocarpa* (Chokeberry) on BMI

**FIGURE 3:**
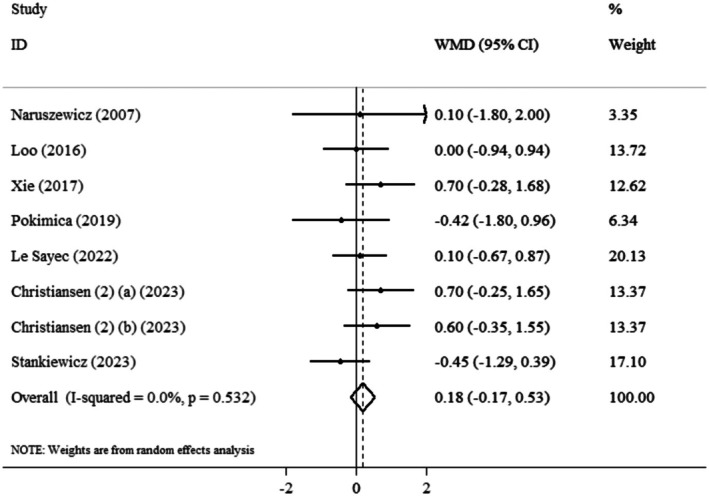
Forest plot detailing weighted mean difference and 95% confidence intervals (CIs) for the effect of *Aronia melanocarpa* (chokeberry) consumption on BMI.

The random effect model illustrated that the pooled mean effect size was not significant (WMD: 0.18 kg/m^2^, 95% CI: −0.17, 0.53, *p* = 0.494) (*I* ^2^ = 0.0%, *p* = 0.304) compared to the control group (Figure 3). Based on findings from a subgroup analysis, *Aronia melanocarpa* did not significantly affect BMI across different age groups, intervention durations, or BMI categories (Table 3).

### 3.5 Effect of *Aronia melanocarpa* (Chokeberry) on WC

**FIGURE 4:**
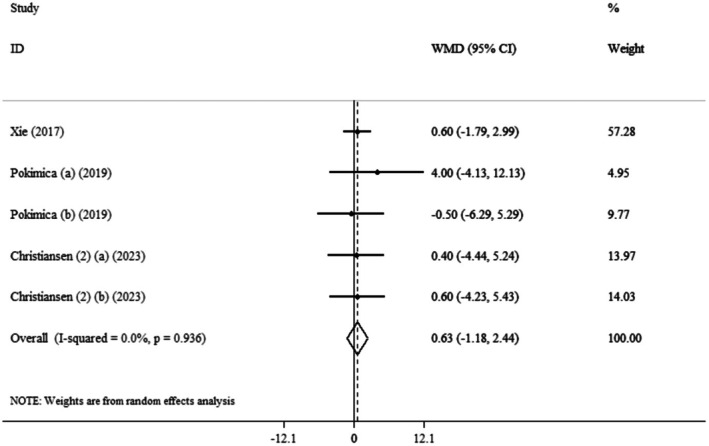
Forest plot detailing weighted mean difference and 95% confidence intervals (CIs) for the effect of *Aronia melanocarpa* (chokeberry) consumption on WC.

This meta‐analysis indicated that the pooled mean effect size was not significant (WMD: 0.63 cm, 95% CI: −1.18 to 2.44, *p* = 0.493) (*I* ^2^ = 0.0%, *p* = 0.936) compared to the control group (Figure 4).

### 3.6 Effect of *Aronia melanocarpa* (Chokeberry) on FBS

**FIGURE 5:**
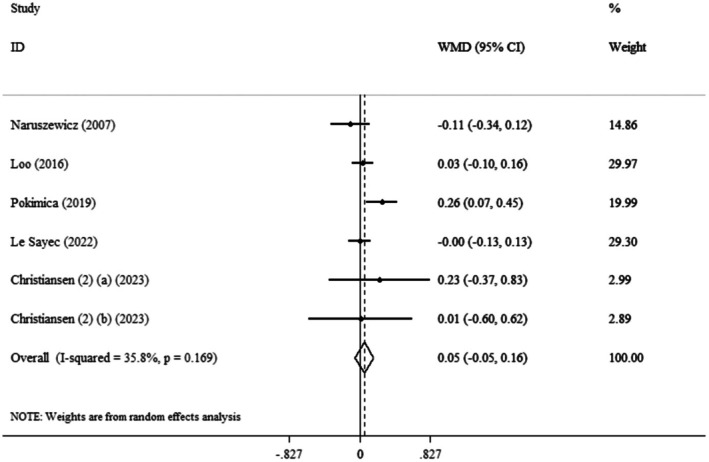
Forest plot detailing weighted mean difference and 95% confidence intervals (CIs) for the effect of *Aronia melanocarpa* (chokeberry) consumption on FBS.

Based on the pooled analysis, the effect of Aronia supplementation on FBS was not significant (WMD: 0.05 mmol/L, 95% CI: −0.05 to 0.16, *p* = 0.341) (*I* ^2^ = 35.8%, *p* = 0.169) compared to the control group (Figure 5).

### 3.7 Sensitivity Analysis

To assess the effect of each trial on the pooled effect size, each study was removed individually from the analysis. The results of the sensitivity analysis showed no influence on the overall estimates when any trial was removed.

### 3.8 Publication Bias

To detect the publication bias, Egger's weighted regression test was performed. The results of Egger's test revealed no publication bias for BW (*p* = 0.133), BMI (*p* = 0.960), WC (*p* = 0.558) and FBS (*p* = 0.770).

## 4 Discussion

The effects of *Aronia melanocarpa* (chokeberry) on BW, WC, BMI, and FBS were evaluated by analysing 7 RCTs. The minimum duration of the studies was 4 weeks, and the maximum duration was 13 weeks. Cochrane criteria were used for the systematic assessment of bias in the included studies. Bias analysis showed that the overall studies included in the quantitative analysis are high‐quality research. The results obtained in the present investigation did not reveal any significant effect of Aronia consumption on the aforementioned variables compared with placebo, according to our pooled data. Also, the subgroup analysis of included RCTs did not indicate any significant effect of *Aronia melanocarpa* (chokeberry) consumption on weight and BMI.

Anthropometric indicators have been used to assess the nutritional situation. The BMI takes into account both height and weight. It is well known that BMI metrics are not always accurate markers of obesity since it is a poor indicator of body fat content. Therefore, WC was also investigated as a measure for managing obesity [8].

The association between the use of Aronia juice/extract and anthropometric measurements seems to be ambiguous. Chokeberry consumption regulates the gene expression of lipogenesis, lipolysis, energy homeostasis and thermogenesis in liver and adipose tissues and could reduce weight gain in mice fed a high‐fat diet [26]. An RCT examining supplementation with a mixture of polyphenol‐rich beverage (PRB) containing 51% chokeberry, cranberry, and pomegranate in 36 healthy men which were randomly divided to consume either 750 mL of a PRB or a placebo drink daily for 8 weeks, found that both groups of participants showed similar trends in body weight and composition over the time course of the study [27]. Other research on people with metabolic disorders reported no effect on anthropometric measurements [19, 25, 28]. In studies with healthy individuals, no effect was observed [29].

It seems that consumption of Chokeberries in a short period (from 4 to 13 weeks) does not affect anthropometric parameters. The overall eating pattern throughout the day and the caloric restriction are very important to lose weight. However, long‐term Aronia may change the adipose tissue energy metabolism by gene expression regulation, which, so far, has only been proven in animal studies.

In the search for preventive programmes and therapeutic goals, the potential role of specific bioactive substances naturally present in or derived from foods to prevent and delay hyperglycemia is to be considered. A certain number of foods contain anthocyanins and their bioactive derivatives. Epidemiological studies have suggested that the consumption of anthocyanins lowers the risk of diabetes and diabetic complications. Anthocyanins may lower blood glucose by improving insulin resistance, protecting β cells, increasing secretion of insulin and reducing digestion of sugars in the small intestine [30]. It has been found that chokeberry contains numerous active ingredients, such as anthocyanins and other polyphenols, and effectively can improve carbohydrate metabolism [8, 31].

It is worthy of note that, animal studies suggested that chokeberry fruit derivatives can have a positive effect on certain metabolic disorders, such as hypoglycemic [32, 33, 34], and hypotensive [35, 36]. Interestingly, chokeberry extract was found to have positive effects on treating type 1 diabetes. In the animal models for T1D, when rats were gavaged daily with an extract of chokeberries (10 or 100 mg/kg) for 1 month, the blood glucose levels were found to be decreased, and the mouse pancreas *β* cells were protected. These results suggest that the consumption of chokeberry may have protective effects on pancreatic *β* cells and in the treatment of T1D [32]. The daily dose of dried Aronia powder corresponded to a total of 150 mg anthocyanins per day, for which no statistically significant effects were observed regarding blood sugar regulation [37]. A systematic review of the clinical trials on Aronia products concluded that daily doses of Aronia preparations should contain between 300 and 600 mg of anthocyanins to achieve significant results [38]. In a study by Lancrajan et al. [39], a 40‐day supplementation with 30 mL of Aronia extract contributed to a decrease in plasma‐sugar levels, from 102.88 mg to 83 mg. Nevertheless, because this trial was performed with only one patient, this paper can only guide the research by pointing to a beneficial property. In addition, there was no data available on the consumption of the overall daily diet and the medications used by the patient. Knowledge regarding the bioavailability of anthocyanins, polyphenol metabolites and metabolism of them following consumption of chokeberry extract is still restricted. Some research studies demonstrate a weak intestinal absorption of the polyphenolic compounds from Aronia juice, even after consumption of a large amount of condensed Aronia juice, which could account for the scant health benefits found in the literature [40, 41].

Natural products offer a robust source for the discovery of therapeutic agents for innovative drugs to support public health, from which the novel structures produced have long been used for the design and discovery of efficient, safe and new therapeutic entities to manage effectively human diseases. Of these, herbs have played a central role in promoting human health historically, and many effective herb‐derived agents have been developed as effective agents for the treatment of cardiometabolic and other diseases over the recent decades. Currently, coronary artery disease, diabetes, obesity, chronic kidney dysfunction, liver disease and cancer are major problems that impair human well‐being. All of these diseases involve oxidative stress that leads to apoptotic cell death and the increased progression of their pathogenesis. Thus, herbs rich in antioxidants can help to prevent and control various types of diseases, owing to their prevention of free radical production that damages normal cells. Phenolic components of plants were found to show effects on human well‐being through antioxidant activity. Aronia berries can have a beneficial effect on health due to their high content of bioactive components, especially polyphenols [8].

Although Chokeberries and their bioactive derivatives have been demonstrated to afford beneficial effects on the prevention and management of disorders associated with oxidative stress, their effectiveness as observed in clinical trial investigations to date seems to be weak. Although polyphenolic compounds have been identified as the vital biological active components of Chokeberries, the antidiabetic, antiobesity, and neuroprotective components of these berries have not been determined obviously [42]. Changes in blood sugar levels were reported by studies in which the intervention was conducted over 10 weeks in patients with disorders of carbohydrate metabolism [8]. Consumption of Chokeberries did not impact anthropometric indicators; however, it seems that Chokeberries can be recommended for many patients with an imbalance of metabolic processes due to the richness of bioactive ingredients [8].

## 5 Limitations

There were some limitations associated with this research. First, a majority of all clinical trials incorporated in this manuscript consisted of a limited number of participants, and the overall count of studies examined was approximately small. Hypothetically, these constraints could result in uncertain evaluations of healing outcomes. Second, many of the incorporated RCTs were short‐term administrations, lasting less than 8 weeks, which may constrain insights into the long‐term effects of Aronia's impact on weight loss and FBS outcomes. Finally, the studies did not consistently control dietary consumption, which may have influenced the outcomes. Furthermore, Chokeberry is tested in 2 different formulations, juice and extract, which have different macronutrient compositions. Unfortunately, the studies do not provide the exact composition. However, Chokeberry extracts probably have a higher content of dietary fibres than juice. Furthermore, the low number of articles in the quantitative analysis made evaluations of publication bias and implementation of sensitivity analyses impossible.

## 6 Conclusions

In conclusion, there are few studies examining Aronia's impact on WC, FBS, BMI, and BW and the results of the present meta‐analysis indicate no effect of Aronia berries consumption on the aforementioned variables. There are a few discrepancies in the studies related to the review in terms of the population being studied, duration of intervention of Aronia, and mode of intervention. Given the lack of agreement provided for certain variables, it is strongly recommended that investigations with appropriately powered sample sizes, and high‐quality RCTs, would enable vagueness to be elaborated. Thus, future studies for Chokeberries could focus more on optimising the doses of phenolic and other constituents present and on new formulation development, as well as the isolation of new active compounds and their synthetic modifications. It seems that large population studies and long‐duration treatment are still needed to draw a clear conclusion about the effect of Aronia berries consumption on the aforementioned variables.

## Author Contributions

N.L.: writing – original draft. M.E.: writing – original draft. M.G.: writing – original draft. N.B.: writing – original draft. F.S.: writing – original draft. F.D.: methodology, writing – original draft. G.A: writing – review and editing. M.R.A.: data curation, formal analysis, methodology, supervision, writing – review and editing.

## Funding

The authors received no funding from an external source.

## Ethics Statement

The authors have nothing to report.

## Consent

The authors have nothing to report.

## Conflicts of Interest

The authors declare no conflicts of interest.

## Supporting information


**Table S1:** Search syntax.

## Data Availability

The data used to support the findings of this study is available from the corresponding author upon request.
