# Supplementary material for: The Effect of Aronia melanocarpa (Chokeberry) on Body Weight and Fasting Blood Sugar: A Systematic Review and Meta‐Analysis of Randomised Controlled Trials
Source: Endocrinol Diabetes Metab. 2025 Nov 29;9(1):e70139. doi: 10.1002/edm2.70139 (PMC12664649; doi:10.1002/edm2.70139)
Supplement: Supplementary file 1 — Table S1: Search syntax. [file EDM2-9-e70139-s001.docx]

**Supplementary Table 1.** Search syntax.

| **Search syntax** | |
| --- | --- |
| (Chokeberry[tiab] OR aronia[tiab] OR "aronia melanocarpa"[tiab] OR Photinia[Mesh]) AND (intervention[tiab] OR RCT[tiab] OR randomized[tiab] OR random[tiab] OR Randomly[tiab] OR Placebo[tiab] OR Assignment[tiab] OR trial[tiab] OR trials[tiab] OR randomised[tiab] OR "Methods"[Mesh] OR Cross-Over[tiab] OR "Double-Blind"[tiab] OR "Randomized Controlled Trial"[Publication Type] OR "Controlled Clinical Trial"[Publication Type] OR "Placebos"[Mesh] OR "Placebo Effect"[Mesh] OR "Clinical Trial"[Publication Type] OR "Clinical Trials as Topic"[Mesh] OR "Cross-Over Studies"[Mesh] OR "Double-Blind Method"[Mesh]) | **Pubmed** |
| ( TITLE-ABS-KEY ( Chokeberry ) OR TITLE-ABS-KEY ( aronia ) OR TITLE-ABS-KEY ( "aronia melanocarpa" ) AND TITLE-ABS-KEY ( intervention ) OR TITLE-ABS-KEY ( "controlled trial" ) OR TITLE-ABS-KEY ( randomized ) OR TITLE-ABS-KEY ( random ) OR TITLE-ABS-KEY ( randomly ) OR TITLE-ABS-KEY ( placebo ) OR TITLE-ABS-KEY ( assignment ) OR TITLE-ABS-KEY ( "clinical trial" ) OR TITLE-ABS-KEY ( trial ) OR TITLE-ABS-KEY ( randomised ) ) AND ( LIMIT-TO ( DOCTYPE , "ar" ) ) AND ( LIMIT-TO ( LANGUAGE , "English" ) ) AND ( LIMIT-TO ( SRCTYPE , "j" ) ) | **Scopus** |
| Chokeberry OR aronia OR "aronia melanocarpa" (All Fields) and intervention OR RCT OR randomized OR random OR Randomly OR Placebo OR Assignment OR trial OR trials OR randomized OR Cross-Over OR "Double-Blind" (All Fields) | **Web of Science** |
| (Chokeberry OR aronia OR "aronia melanocarpa"):ti,ab,kw AND (intervention OR RCT OR randomized OR random OR Randomly OR Placebo OR Assignment OR trial OR trials OR randomized OR Cross-Over OR "Double-Blind"):ti,ab,kw | **Cochrane Library** |
